# First-Principles Investigation of Adsorption of Ag on Defected and Ce-doped Graphene

**DOI:** 10.3390/ma12040649

**Published:** 2019-02-21

**Authors:** Zhou Fan, Min Hu, Jianyi Liu, Xia Luo, Kun Zhang, Zhengchao Tang

**Affiliations:** 1 School of Materials Science and Engineering, University of Southwest Petroleum, Chengdu 610500, China; winfreed@163.com (X.L.); 18782323983@163.com (K.Z.); kt_panda@163.com (Z.T.); 2 State Key Laboratory of Oil and Gas Reservoir Geology and Development Engineering, Southwest Petroleum University, Chengdu 610500, China; liujianyi163@163.com

**Keywords:** first-principles calculation, graphene, Ag adsorption, wettability, defect and Ce doping, Ag-graphene composite filler

## Abstract

To enhance the wettability between Ag atoms and graphene of graphene-reinforced silver-based composite filler, the adsorption behavior of Ag atoms on graphene was studied by first-principles calculation. This was based on band structure analysis, both p-type doping and n-type doping form, of the vacancy-defected and Ce-doped graphene. It was verified by the subsequent investigation on the density of states. According to the charge transfer calculation, p-type doping can promote the electron transport ability between Ag atoms and graphene. The adsorption energy and population analysis show that both defect and Ce doping can improve the wettability and stability of the Ag-graphene system. Seen from these theoretical calculations, this study provides useful guidance for the preparation of Ag-graphene composite fillers.

## 1. Introduction

Silver-based filler and graphene are the two main materials that will be discussed in this study. Silver-based filler is a very important kind of brazing material, which has the advantages of a high strength, good mechanical properties, a high electrical conductivity, corrosion resistance, and so on [1,2,3]. It can join carbide, ceramics, glass, and diamond, and therefore plays an important role in the development of brazing material [4,5,6,7]. Silver-based filler is widely used in the aerospace, super-hard tools, and other manufacturing fields. Owning to its two-dimensional structure and unique physicochemical properties [8,9,10], graphene has excellent properties, such as a high surface area, fracture strength, and low density [11,12], making itself an ideal reinforcement for high performance composites [13,14,15,16]. At present, although graphene reinforced silver-based composite filler has been extensively studied in various types of metal and ceramic welding [17,18,19,20,21,22], it still faces the potential problem of poor wettability for graphene [23]. Experiments have shown that the adsorption energy and charge transfer between Ag and graphene are low due to the poor wettability [24,25,26], whereas doping in graphene can improve the interaction and charge transfer between graphene and metal [27]. Ali Ashraf [28] suggested that the doping-induced modulation of the charge carrier density in graphene influences its wettability and adhesion, for the first time. Recently, the results of some experiments showed that the vacancy defect can increase the adsorption energy and increase the Fermi level [29]. In addition, a further study found the formation of p-type doping with vacancy defect graphene, which can promote the electron transport of graphene and metal [30]. The above studies show that doping can significantly improve the adsorption performance between graphene and metal. Rare earth elements are called metal "vitamins", and trace rare earths can significantly increase the properties of metals [31]. Previous experimental studies have shown that doping with rare earth elements can improve the wettability of Ag30CuZn filler [32]. Based on this finding, the hypothesis has been proposed that doping graphene with rare earth element Ce would enhance the wettability between graphene and Ag. Besides, it is usually difficult to obtain the data of adsorption energy and charge transfer from the experiments. Therefore, a first-principles quantum-mechanical calculation is an effective way to investigate the interaction between different types of atoms.

In this study, the adsorption behavior of Ag atoms on vacancy-deficient graphene (VG) and Ce-doped graphene was calculated by first-principles theory. By analyzing the adsorption energy, band structure, and population, the effects of vacancy defects and Ce doping on the adsorption behavior of Ag on graphene were investigated. The interaction between Ag and graphene and electron transfer were discussed. This research will help to improve the wettability between graphene and silver-based composite fillers, thus providing theoretical guidance and technical support for the application of graphene-reinforced composite fillers.

## 2. Theoretical Method

The calculations were performed using the CASTEP code [33], which is a plane-wave, pseudo potential program based on density functional theory (DFT) [34,35,36]. The electron exchange-correlation interactions were expressed with a generalized gradient approximation (GGA) [37,38] in the form of the Perdew–Burke–Ernzerh (PBE) of functional [39]. The ultrasoft pseudopotential was used to describe the interaction inside and outside the domain approximately. The plane wave kinetic energy cutoff was set to 450 eV, and Brillouin-zone integration was performed with a 3 × 3 × 1 Monkhorst–Pack k-point mesh. The setting of the k point not only ensures the accuracy of the results, but also controls the computational efficiency. The convergence tolerances for the geometric optimization were set as follows: the force on each atom was 0.05 eV/Å; the max stress on each cell was 0.1 GPa; the max displacement on each atom was 0.002 Å; and the convergence energy was 2.0 × 10^−5^ eV/atom.

**Figure 1:**
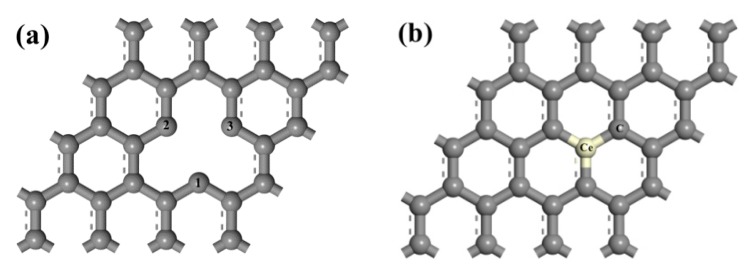
Physical models of graphene: (**a**) VG; (**b**) Ce-doped graphene.

A 4 × 4 supercell with a periodic boundary condition along the x-y plane was employed for the infinite graphene sheet model. The vacuum was set with 20 Å in the z direction, which could avoid the interaction between periodic graphene layers [40]. By removing a chosen C atom in intrinsic graphene, the model of VG was built. The model of Ce-doped graphene was established by substituting a C atom in intrinsic graphene with a Ce atom. To create a model of Ag adsorption on VG or Ce-doped graphene, a single Ag atom was placed above the graphene. Here, we consider the adsorption of the atom on three sites: the hollow (H) site in the center of a hexagon and hollow binding site for vacancy-deficient graphene, the bridge (B) site at the midpoint of a C–C bond and C–Ce bond, the top (T_C_) site directly above a C atom, and the top (T_Ce_) site above the Ce atom. The models of VG and Ce-doped graphene are shown in Figure 1. 

The adsorption energy of an Ag atom on graphene (*E_ad_*) can be computed by

$$E_{ad}=E_{Ag-graphene}-\left(E_{Ag}+E_{graphene}\right)$$

where *E_Ag-graphene_*, *E_Ag_*, and *E_graphene_* represent the total energy of the graphene-Ag system, the energy of the Ag atom, and the energy of graphene, respectively.

## 3. Results and Discussion

### 3.1. Adsorption Energy and Charge Transfer

After structure optimization, it was found that the top-site (T) is energetically favored for Ag atom adsorption in comparison with the adsorption on either hollow (H) or bridge (B) sites in intrinsic graphene and Ce-doped graphene (Table 1). This result is similar to that of Granatier, who calculated interaction energies and geometries of the coronene-X2 complexes d at the MP2 and M06-2X levels, and found that the preferred adsorption site corresponds to a double top-top site [41]. We also found that the hollow site was the most stable adsorption position in the vacancy-deficient graphene because of the lowest adsorption energy (−2.241 eV). Based on this, we only studied the top position of the intrinsic graphene, the T_Ce_ position of Ce-doped graphene, and the hollow binding site for vacancy-deficient graphene in the subsequent calculation. The distance between C2 and C3 decreases from 2.472 Å of intrinsic graphene to 1.809 Å of VG due to the dangling bonds. In addition, local deformation around Ce takes place in Ce-doped graphene due to the difference in bond lengths between Ce–C and C–C. Table 1 shows the final adsorption distance, adsorption energy, and charge transfer between the Ag atom and graphene after Ag adsorption. It was found that due to the formation of the vacancy defect, the adsorption energy between the Ag atom and VG increases from 0.011 eV to 2.439 eV. There is a smaller charge transfer from silver atoms to intrinsic graphene (0.09) in comparison with charge transfer in VG-Ag complexes (0.78). Our results are consistent with Roxana’s finding that the charge is transferred from the Ag atom to graphene sheets in the graphene- silver system [42]. The electronic interaction between Ag and VG is also significantly enhanced, which shows an improvement in wettability. The adsorption height decreases from 1.564 Å of intrinsic graphene to 1.025 Å of VG. For Ce-doped graphene, the charge of Ce lost 1.96e, while the charge of Ag increased by 0.17e. This indicates that electrons are transferred from Ce atoms to graphene, and the adsorption height is also lower than that of intrinsic graphene, which forms chemisorption, and leads to an increase in stability and wettability of the system. It was also revealed that the deformation increases from 0.055 Å of intrinsic graphene to 0.380 Å of VG and 0.147 Å of Ce-doped graphene, representing a large amount of deformation.

**Figure 2:**
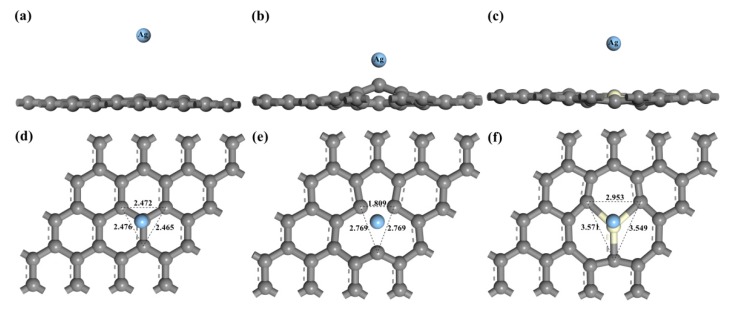
Geometric structures of graphene with the adsorbed Ag atom: (**a**) front view of intrinsic graphene; (**b**) front view of VG; (**c**) front view of Ce-doped graphene; (**d**) top view of intrinsic graphene; (**e**) top view of VG; (**f**) top view of Ce-doped graphene.

**Figure 3:**
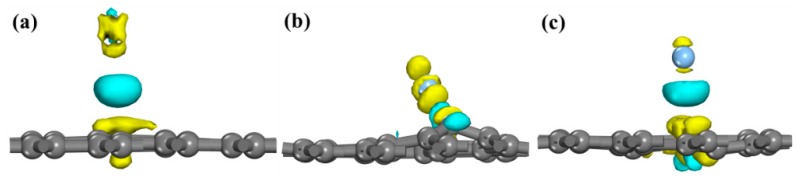
Electron density difference. The yellow and cyan regions represent charge depletion and charge accumulation, respectively. (**a**) Intrinsic graphene; Isosurface level: 0.015 eÅ^−3^; (**b**) VG; Isosurface level: 0.05 eÅ^−3^; (**c**) Ce-doped graphene. Isosurface level: 0.05 eÅ^−3.^

Figure 2 shows the final geometric structures, while Figure 3 indicates the electron density difference of intrinsic graphene, VG, and Ce-doped graphene after adsorption of the Ag atom. The adsorption distance between Ag and graphene decreases due to the existence of vacancies and Ce atoms, while the local deformation around the Ce atom and defects is larger, in which the amount of deformed VG is the largest. Symmetry appears after VG adsorbs Ag atoms, the distance between C1 and C2 increases from 2.46 to 2.769, and C2 and C3 are symmetric about C1, as shown in Figure 2. Like VG, there is symmetry in Ce-doped graphene. C2 and C3 are symmetric about Ce-C1, with the distance between C1 and C2 increasing from 2.471 Å to 3.571 Å of intrinsic graphene. However, the calculated distortion of the intrinsic graphene upon adsorption of the Ag atom was found to be negligible, in agreement with the previous results in Amft [43].

The system change of VG adsorption of Ag atoms is the largest, as shown in Figure 3. The yellow and cyan regions represent charge depletion and charge accumulation. The charge transferred between the Ag atom and the nearest C atom is limited, and the Isosurface level is just 0.015 eÅ^−3^ in Figure 3a. The charge of the Ag atom is mainly transferred to the nearest C atom in the VG system, and there is a bonding pair between Ag and C, forming a stable chemical bond. The Ce atom is in the shape of a petal, and the bond orbital is d-orbital in Ce-doped graphene. The result of charge transfer is the formation of a covalent bond between the Ag atom and the Ce atom, and the Isosurface level is 0.05 eÅ^−3^, which proves that the wettability of the system has been improved.

### 3.2. Density of States

**Figure 4:**
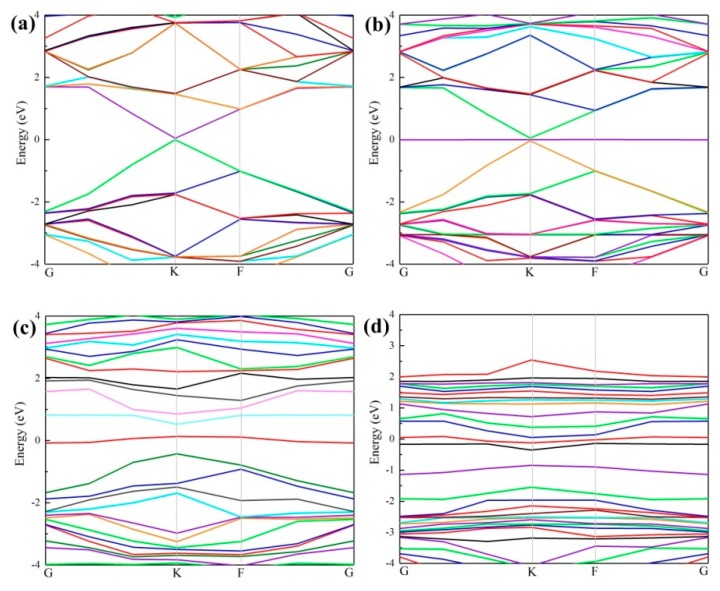
Band structure: (**a**) Intrinsic graphene; (**b**) intrinsic graphene with adsorbed Ag atom; (**c**) VG; (**d**) Ce-doped graphene with adsorbed Ag atom.

To further investigate the mechanism and effect of doping, different band structures were computed, including the intrinsic graphene, VG, and Ce-doped graphene before and after Ag adsorption. The Dirac point [44,45] forms as shown in Figure 4a, which agrees with the experimental data [46]. In Figure 4b, the adsorption of the Ag atom on intrinsic graphene resulted in little change in the band structure. For VG, however, the dangling bonds destroyed the Dirac point [46], resulting in electron deficiency and a slight decline of the Fermi level, as well as movement near the top of the valence band. In this case, p-type doping will form, which promotes a transfer of electrons onto graphene. Figure 4c shows that after the adsorption of the Ag atom on VG, because of the existence of defects, two new energy bands are introduced between the bottom of the conduction band and the top of the valence band. The bottom of the conduction band moves in the high energy direction, while the top of the valence band moves in the low energy direction. The electrons in the Ag atom were transferred to the Fermi level of VG and filled in the electron holes nearby, causing a slight drop of the Fermi level. This result is consistent with the charge transfers in Table 1 (Ag lost 0.78 electrons). Compared with VG, the Fermi level of Ce-doped graphene in Figure 4d experienced an obvious increase, which implied the formation of n-type doping. Since the energy band is relatively flat, the locality is very strong, which infers that the Ce-doping slightly hinders the electron transfer onto the graphene.

**Figure 5:**
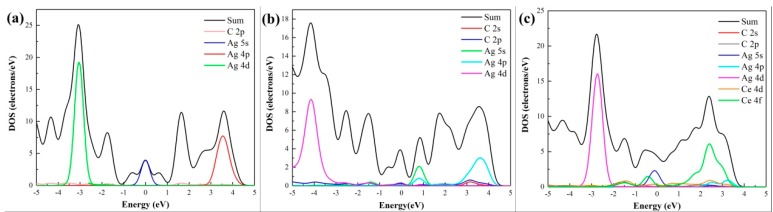
Partial DOS of absorbed Ag atom and its nearest C/Ce atom: (**a**) Intrinsic graphene; (**b**) VG; (**c**) Ce-doped graphene.

The partial density of states (DOS) in Figure 5a shows that, for intrinsic graphene, the d orbital of the adsorbed Ag atom would not interact effectively with the p orbital of its nearest C atom near the Fermi level, confirming that the type of adsorption is physical adsorption, which is consistent with the adsorption energy results in Table 1. LaraCastells [47] found that the Ag2 dimer binds to the surface mostly due to van der Waals-type contributions, which confirms our results. In Figure 5b, both the 4d orbital and 2p orbital of the Ag atom matched well with the 2p orbital of the three dangling C atoms in VG, resulting in a strong coupling [48]. This verifies the formation of a stable chemical adsorption between VG and the Ag atom. In Figure 5c, although the 4d orbital and the p orbital of C atom in graphene display little coupling with the 4d orbital of the Ag atom near the Fermi level, the Ce atom matches well with graphene, suggesting that the adsorption structure is stable in Ce-doping graphene. The results come from the analysis of Figure 5 conformed to the deduction from Figure 4.

### 3.3. Population Analysis

To further study the interaction between graphene and the adsorbed Ag atom, population analysis was completed for intrinsic graphene, VG, and Ce-doped graphene after adsorption of the Ag atom. The results are summarized in Table 2. In the intrinsic graphene–Ag system, there is no population between the Ag atom and the nearest C atom, which shows that the interaction is physical adsorption. In the VG–Ag system, the population between the Ag atom and its nearest C atom is 0.540, and its number of population per unit bond length is 2.561 charge·nm^−1^, indicating that the defect causes chemical adsorption between Ag atoms and graphene, and there is a stable chemical bond, which is consistent with the previous adsorption energy, adsorption height, and DOS analysis. Compared with intrinsic graphene, the population of the Ce-doped graphene-Ag system between the Ce and Ag atoms is 0.28, and its number of population per unit bond length is 0.933 charge·nm^−1^, implying the formation of a stable chemical bond between them.

## 4. Conclusions

Vacancy defects in graphene trigger electron deficiency and p-type doping, which could significantly improve the adsorption energy, charge transfer, and wettability between graphene and the adsorbed Ag atom. Conversely, for Ce-doped graphene, n-type doping formed and thus increased the Fermi level of graphene, which might hinder the electron transfer between Ag and graphene. However, the adsorption energy and population analysis shows that Ce doping could enhance the stability of the Ag–graphene system and wettability.

The adopted research methods, mechanism cognitions, and obtained conclusion in this study might contribute to the future development of efficient and stable brazing materials, and provide useful guidance for the preparation of graphene-reinforced Ag filler with fine properties.

## Figures and Tables

**Table 1:** The final adsorption distance (*D*) and adsorption energy (*E_ad_*) of three bonding sites: top (T), bridge (B), and hollow (H), and charge transfer (*Q*) and deformation (*∆h = h_max_ − h_average_*) after the adsorption of the Ag atom on graphene.

Type of Graphene	D/Å	E_ad_/eV				Q/e	∆h/Å
		T		B	H		
Intrinsic graphene	1.564	−0.011		−0.009	−0.010	0.09	0.055
VG	1.025	−2.358		−2.226	−2.439	0.78	0.380
Ce-doped graphene	1.530	T_Ce_: −2.241	T_C_: −2.230	−2.226	−2.218	Ce: 1.96	0.147
						Ag: −0.17	

**Table 2:** The population between the Ag and its nearest C/Ce atom in graphene.

Adsorption System	Bond	Population	Length/10^−1^ nm	P/charge·nm^−1^
VG	Ag–C1	0.54	2.14657	2.516
	Ag–C2	−0.06	2.51845	−0.238
	Ag–C2	−0.06	2.52454	−0.238
Ce-G	Ag–Ce	0.28	2.99968	0.933
